# UAV-Based Automatic Detection of Missing Rice Seedlings Using the PCERT-DETR Model

**DOI:** 10.3390/plants14142156

**Published:** 2025-07-13

**Authors:** Jiaxin Gao, Feng Tan, Zhaolong Hou, Xiaohui Li, Ailin Feng, Jiaxin Li, Feiyu Bi

**Affiliations:** 1 College of Engineering, Heilongjiang Bayi Agricultural University, Daqing 163319, China; a441380540@163.com (J.G.); houzhaolong@byau.edu.cn (Z.H.); 2 College of Information and Electrical Engineering, Heilongjiang Bayi Agricultural University, Daqing 163319, China; lxh19990420@163.com (X.L.); 19845943454@163.com (A.F.); 18504641963@163.com (J.L.); bifeiyu11@163.com (F.B.)

**Keywords:** PCERT-DETR, UAV, rice seedlings, missing seedlings

## Abstract

Due to the limitations of the sowing machine performance and rice seed germination rates, missing seedlings inevitably occur after rice is sown in large fields. This phenomenon has a direct impact on the rice yield. In the field environment, the existing methods for detecting missing seedlings based on unmanned aerial vehicle (UAV) remote sensing images often have unsatisfactory effects. Therefore, to enable the fast and accurate detection of missing rice seedlings and facilitate subsequent reseeding, this study proposes a UAV remote-sensing-based method for detecting missing rice seedlings in large fields. The proposed method uses an improved PCERT-DETR model to detect rice seedlings and missing seedlings in UAV remote sensing images of large fields. The experimental results show that PCERT-DETR achieves an optimal performance on the self-constructed dataset, with an mean average precision (mAP) of 81.2%, precision (P) of 82.8%, recall (R) of 78.3%, and F_1_-score (F_1_) of 80.5%. The model’s parameter count is only 21.4 M and its FLOPs reach 66.6 G, meeting real-time detection requirements. Compared to the baseline network models, PCERT-DETR improves the P, R, F_1_, and mAP by 15.0, 1.2, 8.5, and 6.8 percentage points, respectively. Furthermore, the performance evaluation experiments were carried out through ablation experiments, comparative detection model experiments and heat map visualization analysis, indicating that the model has a strong detection performance on the test set. The results confirm that the proposed model can accurately detect the number of missing rice seedlings. This study provides accurate information on the number of missing seedlings for subsequent reseeding operations, thus contributing to the improvement of precision farming practices.

## 1. Introduction

Rice is a staple food source for nearly half of the global population [1], and the uniformity of its planting density directly affects both the yield and quality [2]. However, during the sowing or transplanting process, certain areas may experience seedling gaps, which lead to uneven plant distribution in the field, affecting light exposure and nutrient utilization, and reducing the final rice yield. Consequently, the timely detection and precise identification of these areas are imperative for precision agricultural management and yield optimization. Conventional methods for detecting rice seedlings and missing seedlings depend on manual visual inspection, which is laborious and inefficient for farmers. The rapid advancement of image processing technology and artificial intelligence has led to the development of image-based detection methods for rice seedlings and missing seedlings, offering an efficient and accurate solution.

To address these challenges, this study proposes an improved rice seedling detection algorithm based on the RT-DETR architecture. By introducing a Parallel Atrous Convolution module, a Convolution and Attention Fusion Module (CAFM), and an Efficient Multi-Scale Attention (EMA) mechanism, the model achieves the accurate detection of rice seedlings and missing seedlings in complex field conditions. The proposed PCERT-DETR model balances the detection performance and inference speed, making it suitable for large-scale applications in smart farming.

This study’s structure is as follows. Section 2 provides information and data, detailing the algorithm’s structure and implementation. Section 3 presents the experimental results and analysis. Section 4 provides a discussion. Section 5 provides the summary and conclusion.

## 2. Related Work

The existing crop counting techniques can be categorized into two main types: traditional digital image processing-based crop counting and deep learning-based crop counting [3].

### 2.1. Traditional Image Processing-Based Methods

Digital image processing techniques have historically played a significant role in agricultural analysis, especially in crop counting. These methods typically involve preprocessing images, segmenting crops from the background using features such as their color, texture, and shape, followed by morphological operations, skeletonization, contour detection, and corner detection to complete counting. Li et al. [4] used grayscale and HSI transformations to detect cucumbers in greenhouses, while Li et al. [5] applied Fast Normalized Cross-Correlation (FNCC) to detect immature citrus fruits, achieving a detection rate of 84.4%. Ulzii-Orshikh Dorj et al. [6] employed HSV transformation, thresholding, noise removal, and watershed segmentation to count citrus fruits, showing potential in early yield estimation. Fu et al. [7] focused on banana detection in natural scenes using color and texture features, attaining an average detection rate of 89.63%. Liu et al. [8] introduced a color- and shape-based method under various lighting conditions, achieving an F_1_-score of 92.38%. Furthermore, Liu et al. [9] used chain-code-based skeleton optimization for overlapping wheat seedling counting, with an accuracy of 89.94%. Despite their utility, traditional methods are sensitive to lighting and background variations, limiting their performance in complex field environments.

### 2.2. Deep Learning-Based Methods

Deep learning approaches have significantly advanced the crop detection accuracy and robustness. Wang et al. [10] applied YOLOv5 for rice panicle detection, achieving 92.77% accuracy. Li et al. [11] utilized a Capsule Network (CapsNet) for UAV-based rice feature extraction. Liao et al. [12] implemented threshold-based grayscale segmentation, outperforming Otsu and its variants in both performance and computation. In the field of machine learning, Mohamed Marzhar Anuar et al. [13] evaluated CNN models for detecting defective rice seedlings. Ma et al. [14] improved YOLOv8n for pepper detection. Li et al. [15] used an R-CNN for strawberry counting, reporting accuracies of 99.1% (mature) and 73.7% (immature). Fa-Ta Tsai et al. [16] integrated Yolov5m with BoTNet, ShuffleNet, and GhostNet for tomato detection. Yan et al. [17] modified YOLOv5s for daylily detection. Wang et al. [18] proposed YOLOv9-C for tomato recognition, while Sun et al. [19] developed a YOLOv5-CS model for apple detection, highlighting improvements in AP and speed. Liu et al. [20] introduced the MAE-YOLOv8 model for green plum detection in complex orchard scenes. While these methods show promise, they often struggle with detecting small targets like rice seedlings and distinguishing them from background gaps in UAV imagery. Additionally, most models are computationally intensive, hindering real-time deployment in the field. To overcome these limitations, our study proposes a lightweight, multi-scale attention-enhanced PCERT-DETR model based on RT-DETR. It is specifically designed to detect missing seedlings in complex field environments efficiently, offering a new technical foundation for intelligent agricultural systems. A detailed comparison of the latest research on counting and detecting crops using deep learning is depicted in Table 1.

In this study, we propose an enhanced PCERT-DETR algorithm for accurately detecting and counting rice seedlings and missing seedlings in large-scale fields. Based on the RT-DETR framework, the model integrates several key modules to improve the performance: the PAC-APN module captures multi-scale features and reduces redundancy, the CAFM adaptively fuses global and local features using learned spatial weights, and the EMA module captures both short- and long-range dependencies. This approach aims to improve the automation and precision of missing seedling detection and support the development of intelligent agricultural management. This study integrates the RT-DETR architecture with the multi-level attention and fusion module, specifically for detecting missing rice seedlings in complex field environments. Through the multi-scale feature extraction, feature fusion, and fine-grained classification of the degree of absence, a new technical basis is provided for the future precision crop management system.

## 3. Materials and Methods

### 3.1. Image Acquisition

**Figure 1:**
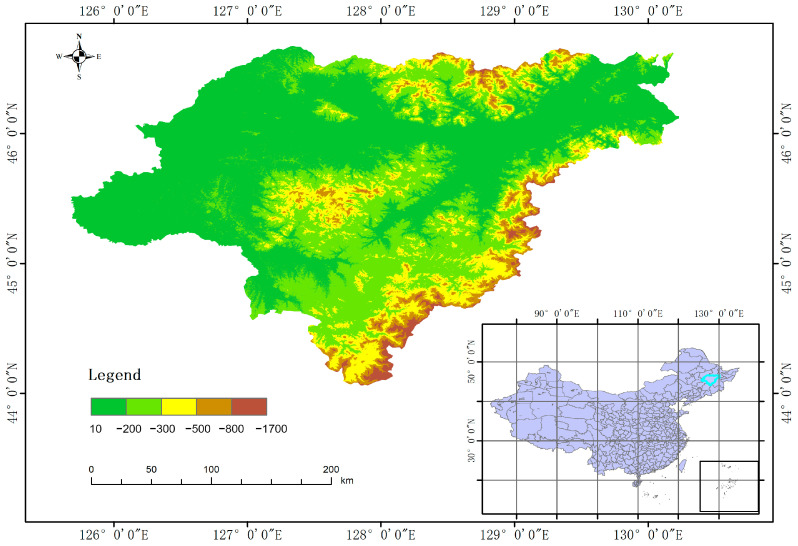
Test site.

**Figure 2:**
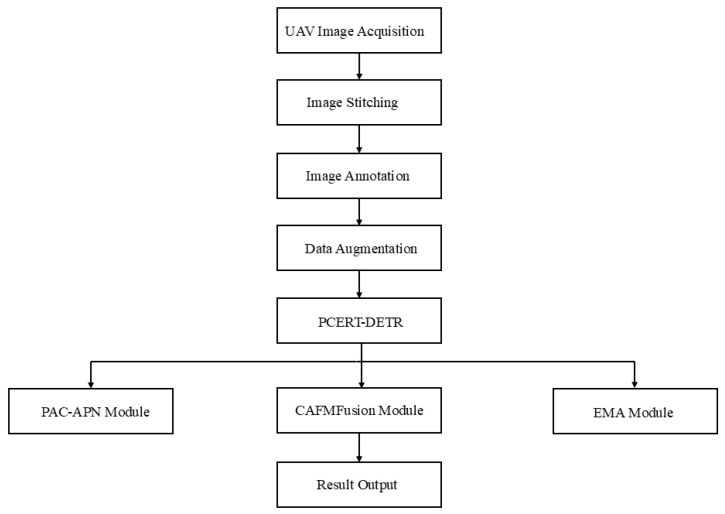
Overall flowchart.

This study concentrated on rice seedlings in cold northern regions, with the UAV image acquisition experiment conducted from 12 June to 20 June 2024. Data collection occurred daily between 10:00 and 13:00 in paddy fields surrounding the Agricultural Internet of Things Service Center in Wuchang City, Harbin, Heilongjiang Province. The geographical coordinates of the study site were determined to be (E 127°18′57.715″, N 45°1′25.597″). The rice cultivar utilized in this study was designated as Daohuaxiang No. 2, with a row spacing of 35 cm and a plant spacing of 20 cm. The data acquisition system employed was the DJI Spirit 3P UAV. The camera’s image sensor was a 1/2.3-inch CMOS with an effective pixel count of 12.4 million (total pixels: 12.76 million). The lens specifications were FOV 94°, 20 mm (35 mm equivalent format), and f/2.8. The camera angle was adjusted to a vertical downward position. The flight speed was set to 2 m per second and the altitude for image capture was 10 m. To prevent a loss of texture feature information in images due to cloud obstruction, data collection was conducted under stable solar radiation intensity and clear, cloud-free sky conditions. During data acquisition, flight paths were planned using Pix4Dcapture (Version 4.4.10), with longitudinal overlap set at 80% and lateral overlap at 70%. The experimental field is illustrated in the following Figure 1. The overall flowchart is shown in Figure 2.

### 3.2. Image Stitching

**Figure 3:**
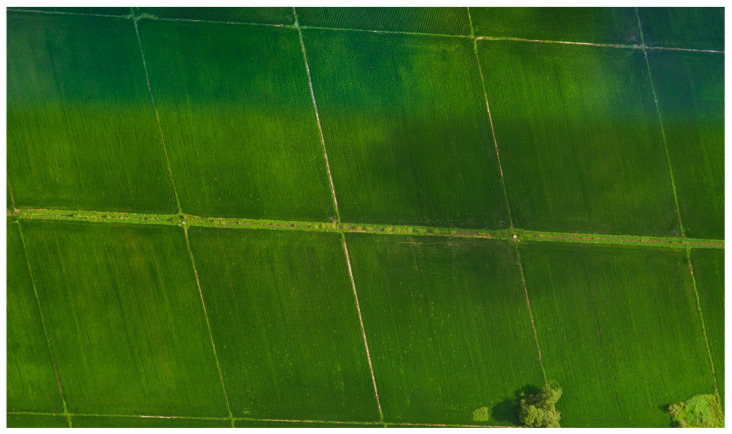
Image stitching.

A total of 600 rice field images were collected in this experiment. The images were then assembled using Pix4Dmapper (Version 4.4.10) to generate a high-resolution map of the study area. The generated orthomosaic is presented in Figure 3.

### 3.3. Data Annotation and Partitioning

**Figure 4:**
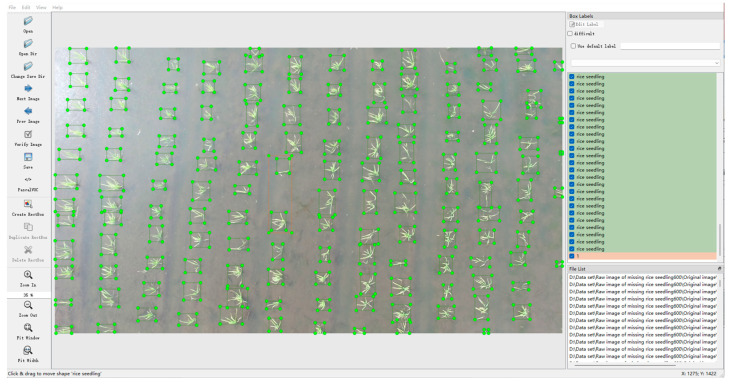
Dataset label distribution.

**Figure 5:**
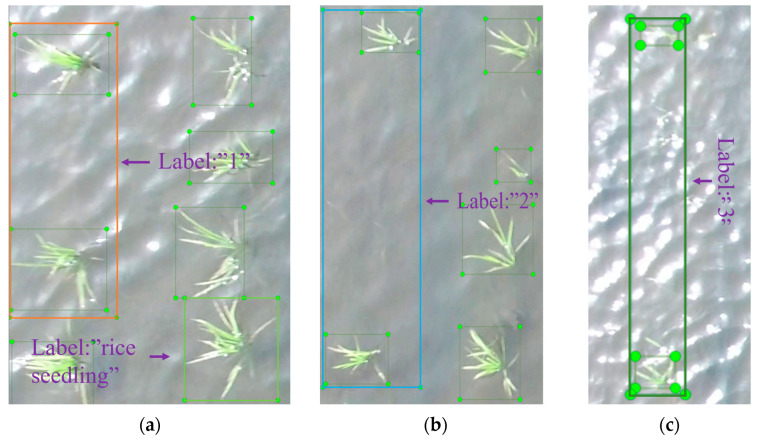
Image labeling: (**a**) seedling labels and missing seedling labels “1”; (**b**) missing seedling labels “2”; (**c**) missing seedling labels “3”.

A total of 600 rice seedling images were obtained in this experiment, with a resolution of 3840 × 2160 pixels. Image annotation was performed using the open-source annotation tool LabelImg (Figure 4), generating XML files containing the width and height of each seedling. The target type was determined by comparing the width and height, distinguishing between rice seedlings and missing seedlings. Rice seedlings were labeled as “rice seedling,” while missing seedlings were annotated with the labels “1,” “2,” and “3,” indicating the number of missing seedlings within the annotation. Among them, the marking of missing seedlings is based on the gap position where “no seedlings appear between the upper and lower pairs of rice seedlings”. Only when there are clearly visible rice seedlings at the adjacent positions above and below the area with missing seedlings will we mark that position as “missing seedlings” to ensure the accuracy of the marking and eliminate the natural spacing caused by non-planting errors. Figure 5 shows the labeled information of the seedlings and the missing seedlings.

**Figure 6:**
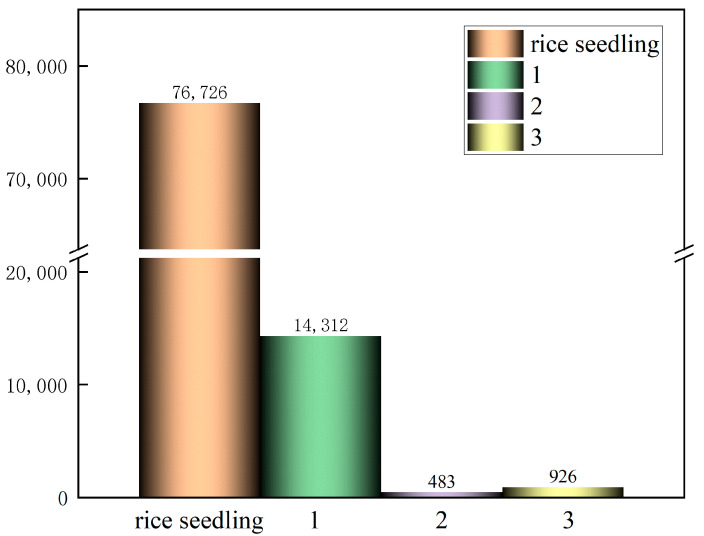
Rice seedling and missing seedling labels.

To facilitate deep learning training, Python(Version 3.10) scripts were used to generate TXT files containing object types and coordinate information. The rice seedling detection and missing seedling detection tasks were annotated within the same dataset. The dataset was randomly partitioned into training, validation, and test sets at a ratio of 7:1:2 (420:60:120 images), respectively, for the purpose of detecting the seedlings. A preliminary analysis of the target labels is illustrated in Figure 6. The dataset contained a total of 76,726 rice seedling labels, 14,312 labels indicating one missing seedling, 483 labels indicating two missing seedlings, and 926 labels indicating three missing seedlings.

### 3.4. Data Augmentation

In the context of deep learning models, data augmentation techniques have emerged as a prevalent approach to enhance the robustness and performance of the model during the training process. This study employs a range of data augmentation techniques on the training set, with the objective of enhancing the model’s robustness for the detection of rice seedling. The augmentation techniques employed included random brightness, contrast adjustments, random shadows, random light spots, random image compression, grayscale conversion, Gaussian noise addition, and simulated weather conditions such as random rain and fog to reflect natural environmental variations.

**Figure 7:**
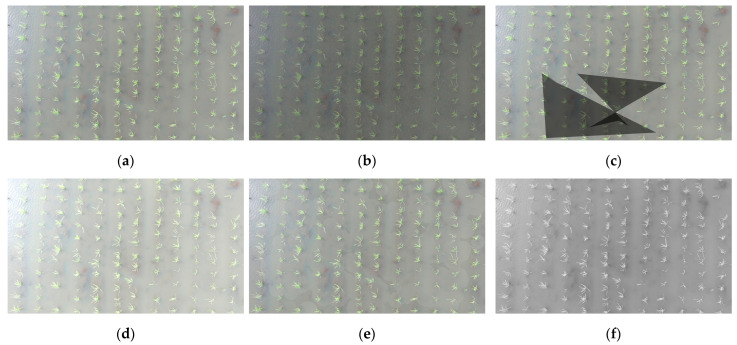
Data augmentation. (**a**) original image; (**b**) random rain; (**c**) random shadows; (**d**) random brightness; (**e**) random light spots; (**f**) grayscale conversion.

During the augmentation process, eight augmentation methods were randomly selected for each image in the dataset, while highly similar augmented images were removed to maintain dataset diversity. After processing, a total of 5350 augmented images were generated. Following the incorporation of these augmented images into the dataset, the final augmented dataset comprised 3745 images in the training set, 1070 images in the test set, and 535 images in the validation set. The efficacy of data augmentation is demonstrated in Figure 7.

### 3.5. Construction of the PCERT-DETR Model

**Figure 8:**
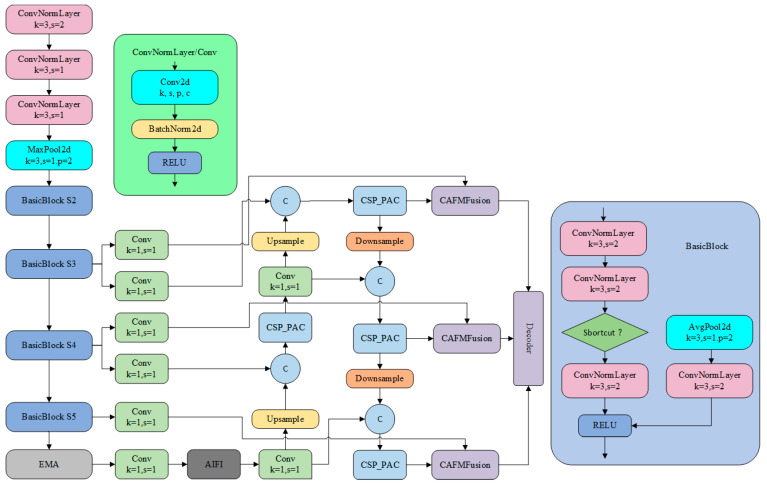
PCERT-DETR model architecture.

In addressing the challenge of detecting missing rice seedlings in expansive fields, where features are frequently indistinct, this study proposes the PAC-APN module. This module captures multi-scale feature information while reducing redundant features, thereby enhancing model performance. Additionally, a hybrid fusion strategy based on CAFM [21] is introduced, which adaptively integrates low-level and high-level features in the encoder by combining global contextual information with local detail information. Learned spatial weights are utilized to adjust feature representations accordingly. Furthermore, the EMA [22] module was engineered with a multi-scale parallel sub-network structure, thereby facilitating the concurrent capture of both short-range and long-range dependencies within images. Through the integration of these three modules into RT-DETR, the enhanced PCERT-DETR model was developed. The network architecture is depicted in Figure 8.

RT-DETR is a real-time object detection framework that integrates two detection approaches: Transformer [23] and DETR [24]. RT-DETR is optimized based on DETR to meet real-time detection requirements, achieving efficient and real-time object detection performance. The PCERT-DETR model proposed in this study is an optimized version of the RT-DETR-r18 model. The design aims to enhance the model’s ability to identify missing rice seedlings in large-scale field environments, allowing it to focus more on detecting missing seedlings and thus improving overall detection performance.

#### 3.5.1. Construction of the PAC-APN Module

In this study, an enhanced model is proposed, founded upon the PAC module, with the objective of enhancing the extraction of features for the purpose of missing rice seedling detection. The PAC module employs parallel atrous convolution layers with differing dilation rates, thereby facilitating the capture of multi-scale features in a simultaneous manner. This addresses the challenge of missing seedling detection in complex paddy field backgrounds. Convolution operations are frequently constrained by their local receptive fields, impeding the capture of global contextual information. In contrast, atrous convolution expands the receptive field without increasing the number of parameters, enabling better multi-scale feature extraction.

(1) $$r_{n}=r_{n-1}+\left(k-1\right)\prod_{i=1}^{n-1}\;s_{i}$$

where $r_{n}$ represents the receptive field of the current layer, $r_{n-1}$ denotes the receptive field of the previous layer, $s_{i}$ represents the stride of the i-th convolutional or pooling layer, and k refers to the standard convolution kernel size.

By replacing the standard convolution kernel k with the dilated convolution kernel $k^{\prime }$, the receptive field calculation formula for dilated convolution can be obtained:

(2) $$r_{n}=r_{n-1}+(k^{\prime }-1)\prod_{i=1}^{n-1}\;s_{i}$$

where $r_{n}$ represents the receptive field of the current layer, $r_{n-1}$ denotes the receptive field of the previous layer, $s_{i}$ represents the stride of the i-th convolutional or pooling layer, and $k^{\prime }$ denotes the standard convolution kernel size.

**Figure 9:**
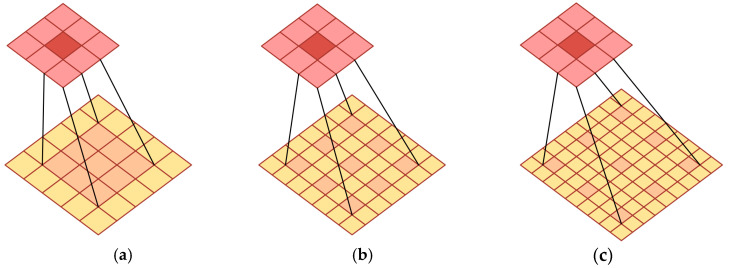
Dilated convolution with different dilation rates. (**a**) Dilation rate = 1; (**b**) Dilation rate = 2; (**c**) Dilation rate = 3.

However, a single dilation rate in atrous convolution may not adequately capture features at all scales. Therefore, a parallel structure that combines multiple atrous convolution layers with different dilation rates is employed. Dilated convolution with different dilation rates is shown in Figure 9 below. This allows the network to simultaneously extract both local detail information and global contextual information. In the task of detecting missing rice seedlings, variations in the seedling morphology and size may arise due to growth stages, lighting conditions, and occlusions. The proposed approach, termed the PAC module, has been shown to enhance detection by capturing intricate leaf edge and texture details while preserving the spatial relationships between plants.

**Figure 10:**
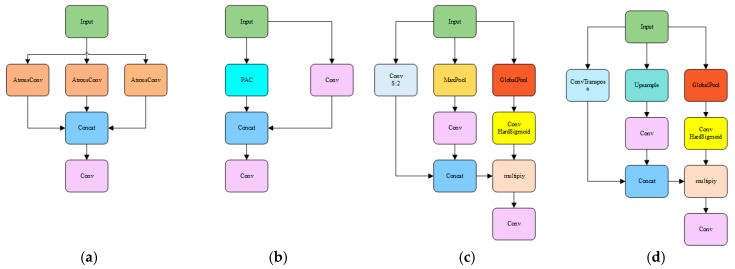
PAC-APN structure. (**a**) PAC; (**b**) CSP_PAC; (**c**) Attention downsample; (**d**) Attention upsample.

In addition, a parallel upsampling/downsampling branch structure is introduced. This design provides multiple feature extraction pathways, thereby significantly enhancing the diversity of feature representations. The upsampling branch focuses on capturing fine details in images, while the downsampling branch extracts global features. This multi-scale feature extraction mechanism enables a comprehensive understanding of the input images, thereby improving detection accuracy. A gating mechanism further optimizes feature selection by dynamically weighting features extracted from parallel branches. The PAC-APN structure is shown in Figure 10. This adaptive feature selection strategy strengthens features relevant to missing rice seedling detection while suppressing redundant or irrelevant features. Consequently, the effectiveness of feature representation is improved, and the model’s robustness in complex farmland environments is enhanced. (See Figure 9 and Figure 10).

#### 3.5.2. Design of the CAFM 

**Figure 11:**
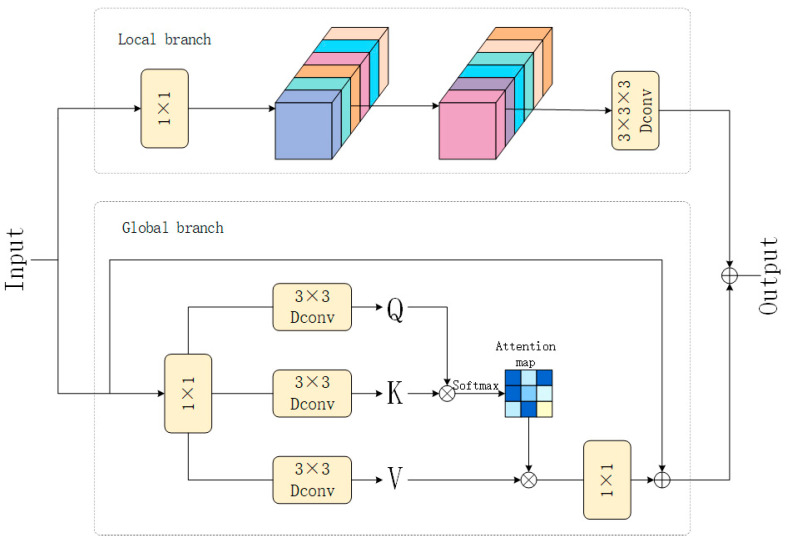
Schematic diagram of CAFM.

In this study, we propose a novel approach for integrating low-level features from the encoder with high-level features, leveraging a hybrid fusion scheme that utilizes the CAFM. This approach adaptively integrates these features by learning spatial weights for feature modulation. CAFM is designed to fuse global and local features, aiming to capture long-range dependencies. The CAFM consists of two branches. The first branch, the local branch, employs a 1 × 1 convolution to adjust the channel dimension, followed by a channel shuffle operation to enhance inter-channel information integration. The channel shuffle operation divides the input tensor into multiple groups along the channel dimension, applying depthwise separable convolution within each group to achieve channel mixing. Finally, the output tensors of all groups are concatenated along the channel dimension to generate the final output. A 3 × 3 × 3 convolution is then applied to extract features. The schematic diagram of CAFM is shown in Figure 11. The local branch can be formulated as:

(3) $$F_{conv}=W_{3\times 3\times 3}(CS(W_{1\times 1}(Y)))$$

where $F_{conv}$ represents the output of the local branch, $W_{1\times 1}$ denotes the 1 × 1 convolution, $W_{3\times 3\times 3}$ represents the 3 × 3 × 3 convolution, CS indicates the channel shuffle operation, and Y is the input feature.

The second branch, the global branch, generates the query (Q), key (K), and value (V) matrices using a 1 × 1 convolution and a 3 × 3 depthwise separable convolution, resulting in three tensors of shape $\hat{H}\;\times \;\hat{W}\;\times \;\hat{C}$. The query tensor Q is then reshaped into $\hat{Q}\in R^{\hat{H}\hat{W}\times \hat{C}}$, and the key tensor K is reshaped into $\hat{K}\in R^{\hat{C}\times \hat{H}\hat{W}}$. The attention map $A\in R^{\hat{C}\times \hat{C}}$ is computed through the interaction between $\hat{Q}$ and $\hat{K}$, reducing computational complexity. The output of the attention mechanism, $F_{att}$ is defined as:

(4) $$F_{att}=W_{1\times 1}Attention\left(\hat{Q},\hat{K},\hat{V}\right)+Y$$

(5) $$Attention\left(\hat{Q},\hat{K},\hat{V}\right)=\hat{V}Softmax\left(\hat{K}\hat{Q}/\alpha \right)$$

where α is a learnable scaling parameter used to control the magnitude of the matrix multiplication before applying the softmax function.

Finally, the output of the CAFM is computed as:

(6) $$F_{out}=F_{att}+F_{conv}.$$

**Figure 12:**
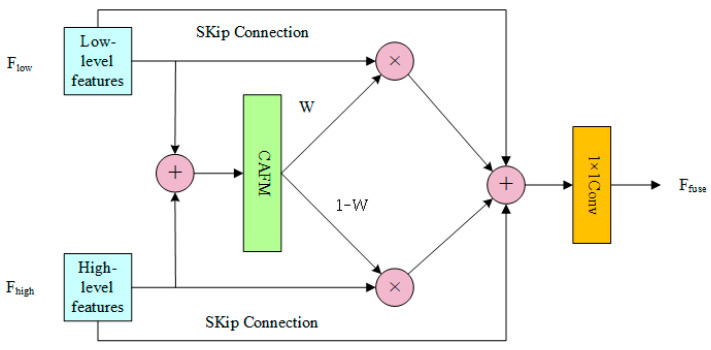
Schematic diagram of fusion.

The CAFM hybrid fusion module has been demonstrated to facilitate more comprehensive capture of key features related to missing seedlings in rice fields through integration of global contextual information with local details. Concurrently, the module has been shown to suppress background noise and interference, thereby enhancing detection performance, schematic diagram of fusion is shown in Figure 12. (See Figure 11 and Figure 12).

#### 3.5.3. The Efficient Multi-Scale Attention Mechanism

**Figure 13:**
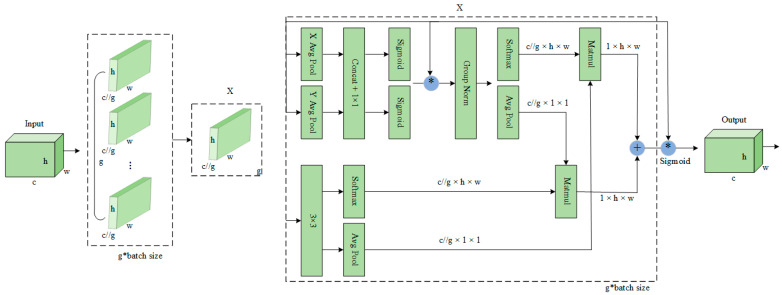
Illustration of EMA. Here, “g” means the divided groups, “X Avg Pool” represents the 1D horizontal global pooling and “Y Avg Pool” indicates the 1D vertical global pooling, respectively.

In this study, we introduced the EMA mechanism as a means to enhance the rice seedling missing detection model. The primary objective of this integration was to improve the model’s ability to perceive multi-scale features and enhance the robustness of feature representation. The EMA model processes input features by dividing them into groups along the channel dimension and employing two parallel branches: one branch uses 1D global pooling and 1 × 1 convolution to capture global information, while the other branch applies 3 × 3 convolution to extract local spatial features. The outputs of both branches are fused through matrix multiplication to generate an attention map, which is then combined with the input features. The EMA structure is shown in Figure 13. This approach enhances the model’s capability to capture both global and local information while reducing computational complexity.

The effectiveness of the EMA module’s multi-scale parallel sub-network design in capturing both short-range and long-range dependencies in images is well-documented. Rice field images, for instance, typically contain complex background information, such as soil, weeds, and shadow interference, while missing seedlings often manifest as local feature absences or irregular distributions. The multi-scale parallel sub-network structure enables the model to extract rich feature representations across different scales, thereby improving the identification of missing seedling areas. The model’s ability to discern short-range dependencies, such as the absence of individual seedlings, is bolstered by long-range dependencies, which facilitate the recognition of large-scale patterns of missing seedling. This multi-scale feature learning capability enhances the model’s adaptability to complex scenarios and improves detection accuracy.

Furthermore, the EMA module reorganizes the channel dimension to prevent information loss caused by conventional channel dimensionality reduction methods. Conventional methods reduce computational costs by decreasing channel dimensions; however, they often lose critical feature information, which is particularly detrimental to rice seedling missing detection where subtle feature differences can be key to identifying missing areas. The EMA module mitigates this issue by redistributing certain channels into the batch dimension, thus reducing computational costs while preserving complete feature information for each channel. This design ensures sufficient and accurate feature representation, allowing the model to efficiently process high-dimensional feature data while retaining crucial information. Compared to existing attention mechanisms, the EMA module significantly enhances detection performance while substantially reducing the number of parameters and computational overhead. (See Figure 13).

## 4. Results and Analysis

### 4.1. Experimental Environment

This test is conducted using the PyTorch framework (version 1.10.0), with Table 2 detailing the experimental environment. The input image size is set to 640 × 640 pixels. The model hyperparameters are configured as follows: the batch size is 16, the SGD optimizer is used with an initial learning rate of 0.01, and the momentum parameter is set to 0.937. The learning rate is adjusted using the cosine annealing decay algorithm, with a decay coefficient of 0.0005. The total number of training iterations is 300. The weight file is saved every 50 epochs, and a log file is generated to record the loss values for both the training and validation sets. These hyperparameters are carefully chosen to ensure faster convergence, reduce overfitting, and avoid the model from becoming stuck in local minima.

### 4.2. Model Evaluation Index

To objectively evaluate the model’s performance in detecting rice seedlings, metrics, e.g., the P, R, harmonic average F_1_-score value, average precision (AP), mAP, the number of network parameters, and FLOPs, are used. In the experiment, the IOU value was set to 0.5. The calculation formulas for the P, R, and F_1_-score are given by Equations (7)–(9).

(7) $$Precision=\frac{TP}{TP+FP}$$

(8) $$Recall=\frac{TP}{TP+FN}$$

(9) $$F_{1}=\frac{2\cdot Precision\cdot Recall}{Precision+Recall}$$

In this study, TP refers to the number of correctly detected objects, FP refers to the number of incorrectly detected objects, FN refers to the number of objects that were not detected by the algorithm, and F_1_ represents the harmonic mean of the precision and recall. When F_1_ is close to 1, it indicates good model optimization. AP refers to the area under the precision–recall (PR) curve, which is formed by the PR curve and the axes. Higher AP values indicate the better performance of the object detection algorithm. mAP represents the average of the AP values across multiple categories, reflecting the algorithm’s overall detection performance across different categories. In this study, mAP is the average of the AP values for rice seedlings, missing one rice seedling, missing two rice seedlings, and missing three rice seedlings, as shown in Formula (10).

(10) $$mAP=\frac{1}{k}\sum_{i=1}^{k}AP_{i}$$

where N represents the number of categories. To assess the model complexity, the number of network parameters and FLOPs are used as parameters for evaluating the model complexity.

### 4.3. Ablation Test Results

**Figure 14:**
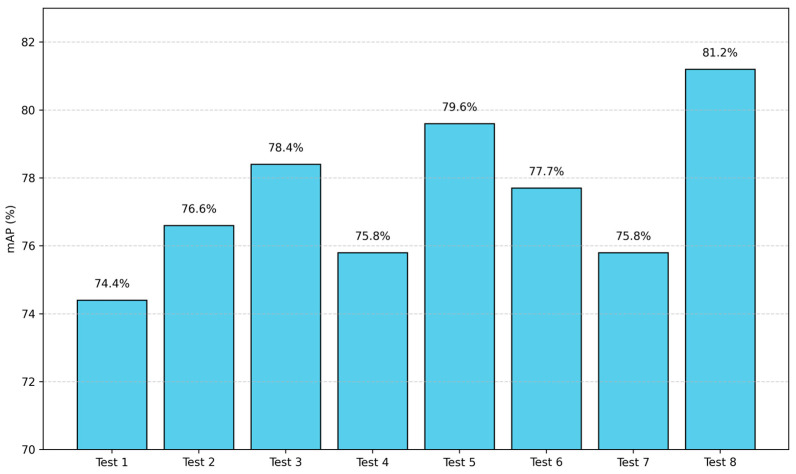
Comparison of mAP across ablation models.

In order to verify the effectiveness of the PACAPN module, CAFMFusion module, and EMA mechanism in the identification of rice seedlings and missing seedlings, an ablation study was conducted using a self-constructed rice image dataset. The comparative results are presented in Table 3. The comparison of the mAP values of each model is shown in Figure 14.

As demonstrated in Table 3, Experiment 2 incorporated the PACAPN module in comparison to Experiment 1, exhibiting no substantial augmentation in the number of parameters, but a notable rise in FLOPs. The precision metric witnessed a 15.1 percentage point escalation, while the recall metric exhibited a modest decline, culminating in a 4.6 percentage point enhancement in the F_1_-score and an aggregate increase in mAP. This suggests that the PACAPN module does not substantially impact the model parameters. The incorporation of parallel atrous convolution layers with varying dilation rates facilitates the multi-scale feature extraction of absent seedlings, thereby enhancing the detection accuracy. In Experiment 3, the CAFMFusion module was integrated into the model in comparison to Experiment 2. While the number of model parameters and FLOPs increased by 1.5 M and 1.2 G, respectively, the P, R, F_1_, and mAP improved by 1.6, 5, 3.5, and 1.8 percentage points, respectively. This enhancement can be ascribed to CAFMFusion’s capacity to amalgamate global and local information, thereby facilitating a more exhaustive depiction of varied field conditions in rice seedling detection, thus enhancing the model’s feature representation capability. In Experiment 4, CAFMFusion was the sole addition, and its performance was found to be inferior to that of Experiment 3 across all evaluation metrics, while FLOPs decreased by 1.3 G. In Experiment 5, which was compared to Experiment 1, there were no significant changes in the parameters and FLOPs. However, P, F_1_, and mAP improved by 14.9, 6.7, and 5.2 percentage points, respectively, while R decreased by 2.1 percentage points. This finding indicates that the EMA attention mechanism, facilitated by a parallel multi-branch structure, extracts features across diverse scales. Consequently, the model is able to comprehensively capture critical features that were previously missing, thereby enhancing the detection accuracy. In Experiment 6, the issue of low recall was effectively alleviated, with a maximum R of 78.5 being achieved. However, other evaluation metrics demonstrated varying degrees of decline. In Experiment 7, the EMA attention mechanism was incorporated into Experiment 2, yet no substantial changes in the parameters and FLOPs were observed. However, a decline was observed in P, R, and mAP by 4.1, 7.1, and 0.8 percentage points, respectively, while F_1_ demonstrated an increase of 1.9 percentage points. The PCERT-DETR model demonstrated the highest level of mAP, attaining 81.2%, which surpassed the RT-DETR model in P, R, F_1_, and mAP by 15.0, 1.2, 8.5, and 6.8 percentage points, respectively. It is noteworthy that F_1_ and mAP attained their maximum values at 80.5 and 81.2, respectively. Despite the augmentation of the model parameters by 1.5 M and FLOPs by 9.6 G, a substantial enhancement in the detection accuracy was observed, underscoring the model’s augmented capacity for feature extraction in the context of missing rice seedling detection.

### 4.4. Detection Model Comparison Test

**Figure 15:**
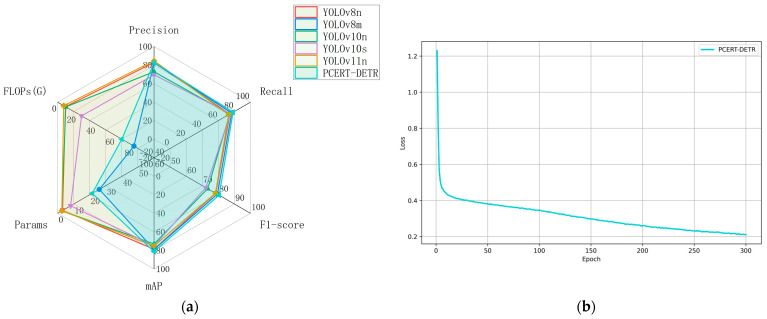
Performance comparison chart of different mainstream standard object detection models. (**a**) Performance comparison chart of precision, recall, F_1_-score, mAP, Params, and FLOPs. (**b**) Training loss.

In order to further evaluate the performance of the proposed PCERT-DETR model, a comparison was made between it and commonly used YOLO series models using a self-constructed missing rice seedling image dataset. The comparison metrics included the Precision, Recall, F_1_-score, mAP, FLOPs, and the total number of parameters. The results are summarized in Table 4. A radar chart analysis based on the data in Table 4 is presented in Figure 15a, while the training loss curve of the PCERT-DETR model is shown in Figure 15b.

A comparison of the PCERT-DETR model and the YOLOv8n model reveals that the former attains a comparable P, while the latter exhibits a 3.9% decline in R, a 2.1% decline in F_1_, and a 2.5% decline in mAP. However, the YOLOv8n model demonstrates a marked reduction in computational costs, with a decrease of 18.3 M in Params and 58.5 G in FLOPs. The YOLOv8m model exhibits a 0.4% decrease in P, a 1.6% decrease in R, a 1.1% decrease in F_1_, and a 2.3% decrease in mAP compared to the PCERT-DETR model. However, the YOLOv8m model demonstrates an increase in computational costs, with Params and FLOPs increasing by 4.5 M and 12.5 G, respectively. The YOLOv10n model demonstrates a decline in the precision, recall, F_1_-score, and mAP of 10%, 4.2%, 7.1%, and 8.2%, respectively, in comparison to PCERT-DETR. Concurrently, its parameters and FLOPs undergo a substantial reduction of 18.7 M and 58.2 G, respectively, thereby enhancing its computational efficiency. A similar trend is observed in the YOLOv10s model, which demonstrates reductions in the P, R, F_1_, and mAP of 13.3%, 3.3%, 8.3%, and 6.1%, respectively, when compared to PCERT-DETR. Additionally, its Params and FLOPs are reduced by 13.3 M and 41.8 G, respectively. Among all the models that were evaluated, the YOLOv11n model demonstrated the highest precision at 84.5%, which is 1.7% higher than the PCERT-DETR model. However, its recall, F_1_-score, and mAP were 5.5%, 2.3%, and 6% lower, respectively. Notably, the YOLOv11n model exhibits the smallest parameter count (2.6 M) and FLOPs (6.3 G), which are 18.8 M and 60.3 G lower than those of PCERT-DETR, respectively.

A comparison of the PCERT-DETR model with other object detection models reveals that, while it does not attain the smallest Params and FLOPs, it exhibits a marked superiority in accuracy metrics, with a mAP of 81.2%, while concurrently satisfying real-time detection requirements. These findings substantiate the pre-eminent overall performance of the proposed PCERT-DETR model in detecting rice seedlings and missing seedlings in the dataset.

### 4.5. Simulation Test

**Figure 16:**
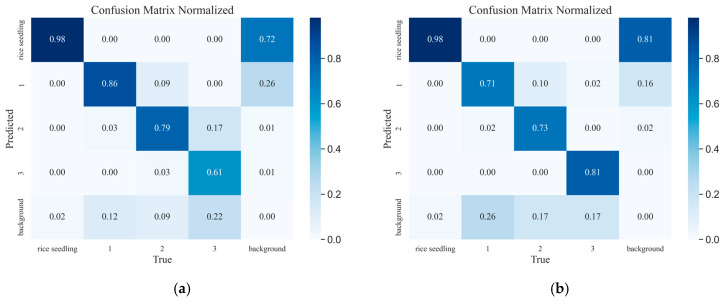
Confusion matrix. (**a**) RT-DETR; (**b**) PCERT-DETR.

In the evaluation of deep learning models, the confusion matrix [25] plays a pivotal role in assessing the classification performance. This matrix, presented in a tabular form, has rows representing the predicted categories and columns representing the actual categories. Each element in the matrix quantifies the number of samples classified into a specific category, with diagonal elements indicating correctly classified samples and off-diagonal elements denoting misclassification cases. The analysis of the confusion matrix provides a comprehensive assessment of the model’s classification performance, offering further validation of its effectiveness. In this study, a comparative analysis was conducted on the classification performance of the model before and after improvement for rice seedling and missing seedling detection. The analysis utilized confusion matrices, as illustrated in Figure 16.

As illustrated by the confusion matrix in Figure 16a, in the test set of the self-constructed missing rice seedling image dataset, the RT-DETR model correctly identified 98% of rice seedlings, while 2% of rice seedlings were misclassified as background. The classifier correctly identified 86% of single missing seedlings, with 3% misclassified as two missing seedlings and 12% misclassified as background. The model demonstrated 79% accuracy in classifying two missing seedlings, with 9% misclassified as single missing seedlings, 3% as three missing seedlings, and 9% as background. For three missing seedlings, the classifier correctly identified 61%, while 17% were misclassified as two missing seedlings and 22% as background. As illustrated in Figure 16b, the PCERT-DETR model attained classification accuracies of 98% for rice seedlings, 71% for single missing seedlings, 73% for two missing seedlings, and 81% for three missing seedlings in the missing rice seedling test set.

The preceding analysis demonstrates that the RT-DETR and PCERT-DETR models attain equivalent classification accuracy for rice seedlings. The RT-DETR model demonstrates superiority in detecting single and two absent seedlings, while the PCERT-DETR model exhibits superiority in detecting three absent seedlings, with a 20% enhancement over RT-DETR. The final column of the confusion matrix displays the false positives (FP) for the background class, with values of 81% for rice seedlings, 16% for single missing seedlings, and 2% for two missing seedlings. It is noteworthy that the highest value of 81% is observed for rice seedlings, indicating that some rice seedling regions with blurred boundaries were omitted.

### 4.6. Feature Visual Analysis

**Figure 17:**
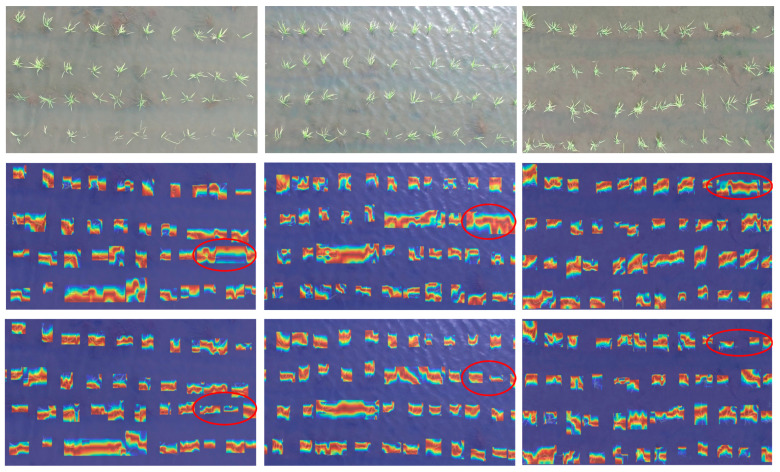
Grad-CAM visualization.

To compare the detection performance of the PCERT-DETR model with the original RT-DETR model on the rice dataset, this paper utilizes Grad-CAM [26] to obtain the attention distribution of different categories during model inference. Grad-CAM provides an intuitive visualization of the specific layer structures of various networks, enabling the further analysis of the model detection effectiveness. The trained PCERT-DETR and RT-DETR models were tested on a subset of the test set. Grad-CAM visualizations were generated for the images in this subset, as shown in Figure 17. The red-circled areas represent differences in the detection results between the two models. From the figure, it can be observed that both models accurately detect rice seedlings through Grad-CAM visualization. However, by examining the red-circled regions, we can see that RT-DETR still exhibits certain errors in detecting missing seedlings. In the first image, RT-DETR mistakenly identifies rice seedlings with minimal water exposure as missing seedlings. In the second image, RT-DETR also exhibits false detections and omissions in the brighter regions. In the third image, RT-DETR incorrectly classifies seedling areas as missing seedling regions. In contrast, our proposed model is capable of detecting less obvious and overly bright seedling areas, as well as missing seedling conditions. PCERT-DETR’s Grad-CAM visualization places greater emphasis on different categories of missing rice seedlings, enabling the model to more accurately capture key features of each category. The analysis of Grad-CAM results demonstrates that the proposed method improves the accuracy and effectiveness of RT-DETR in detecting missing rice seedlings.

### 4.7. Correlation Study of Seedling Leakage Count

**Figure 18:**
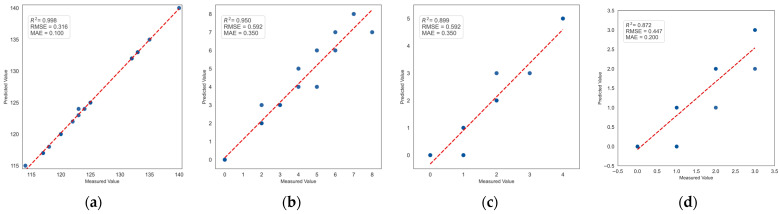
Fitting results of rice seedling and missing seedling count values with actual values. (**a**) Rice seedlings; (**b**) missing one rice seedling; (**c**) missing two rice seedlings; (**d**) missing three rice seedlings.

**Figure 19:**
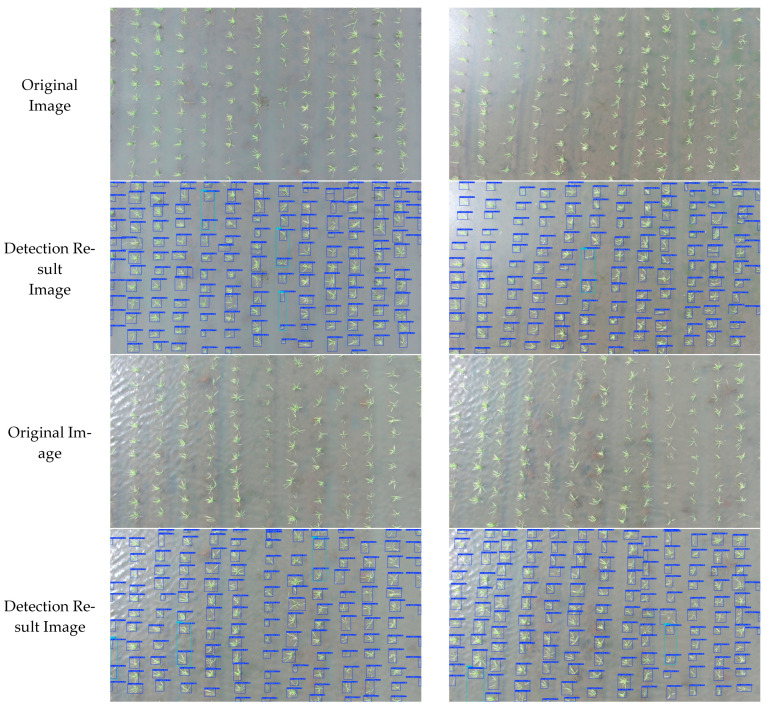
Detection results of some images.

The rice seedlings and missing seedlings present in each image were determined based on the detection images obtained. The estimated number of rice seedlings and missing seedlings from this study was then compared with the actual values, and linear regression was performed to analyze the results. A subset of 20 rice images from the test set was selected for the experiment, and 10 manual counts were conducted for each image. The average value was then taken as the actual value of the rice seedlings and missing seedlings. The fitting results of the estimated values and actual values for rice seedlings and missing seedlings are shown in Figure 18. According to the fitting results, the coefficient of determination (R^2^) values were 0.998, 0.950, 0.899, and 0.872, and the RMSE values were 0.316, 0.592, 0.592, and 0.447, with MAE values of 0.100, 0.350, 0.350, and 0.200, respectively. These findings suggest a clear linear correlation between the estimated and actual values of rice seedlings and missing seedlings. The true number of rice seedlings counted in this study was 2510, with 61 one seedlings missing, 24 with two missing, and 19 with three missing. The rice seedling counts generated by the PCERT-DETR model were 2512, with 64 one missing seedlings, 23 with two missing, and 15 with three missing. The error counts were 2, 3, 1, and 4, respectively, indicating that the proposed model exhibited a low level of error in the counting of rice seedlings and missing seedlings in the images. Figure 19 presents the detection results for rice seedlings and missing seedlings in several images. As illustrated in Figure 19, the rice seedling and missing seedling counts derived from the PCERT-DETR model closely align with the actual counts, thereby substantiating the reliability of the methodology proposed in this study.

## 5. Discussion

### 5.1. Model Performance

In order to validate the performance of the enhanced PCERT-DETR model in detecting rice seedlings and missing seedlings in field environments, a series of comparative experiments, ablation experiments, and visual analyses were conducted. The comparative experiments demonstrated that the PCERT-DETR model achieved the highest mAP. Although the model’s parameters and FLOPs were not the lowest, the speed loss was acceptable, and the model’s mAP was significantly higher than that of other models. The inference speed also met real-time requirements. The confusion matrix revealed that the accuracy of rice seedling detection using the proposed method in this study reached 98%. Compared to a method based on YOLOv4 proposed by Jui-Feng Yeh [27], the AP was improved by 6.26%. In comparison with the models for rice seedling detection in the study by Hsin-Hung Tseng [28], such as HOG-SVM, EfficientDet, and Faster R-CNN, which achieved AP values of 70.2%, 83.2%, and 88.8%, respectively, on the test set, the AP improved by 27.8%, 14.8%, and 9.2%, respectively. In contrast to models that are exclusively designed for rice seedling counting, this study integrates a dual approach that encompasses both seedling detection and the identification of missing seedlings. This comprehensive approach effectively overcomes the limitations inherent in single-counting models when applied in practical field settings.

### 5.2. Practical Implications and Deployment Considerations

While the proposed PCERT-DETR model demonstrates a strong performance in terms of the detection accuracy, its significance extends beyond numerical metrics. In real-world agricultural scenarios, the accurate and timely detection of missing seedlings plays a crucial role in precision farming and yield optimization. By identifying both the location and severity of missing seedlings, the model enables targeted replanting decisions, ensuring a uniform crop density and minimizing the yield loss due to early-stage planting deficiencies.

Moreover, early detection empowers farmers to take timely interventions during critical growth periods, such as re-sowing, pest control, or fertilizer adjustment, thus supporting proactive crop management strategies. Compared to manual field surveys, the use of UAVs combined with PCERT-DETR offers scalable, consistent, and labor-efficient monitoring, which is especially beneficial in large-scale or labor-constrained agricultural systems.

In terms of the hardware applicability, the PCERT-DETR model has been optimized to strike a balance between the precision and computational cost. Inference speed tests indicate that the model can achieve a near real-time performance on edge computing devices equipped with mid-range GPUs, making it suitable for on-site UAV-based detection workflows. For high-throughput batch processing, it can also be deployed on cloud-based platforms or farm-level servers. The modular architecture allows further compression or pruning for lightweight adaptation, enabling flexibility across different deployment environments. The PCERT-DETR model is not only effective in its detection accuracy, but also practical for field-level deployment and decision support in modern precision agriculture.

### 5.3. Limitations and Solutions

The method proposed in this paper has some limitations, mainly in the following three aspects:

(1) The mAP of the PCERT-DETR model proposed in this study is 81.2%, indicating the presence of a detection error. The primary reason for this is the relatively small number of missing seedling samples in the training dataset, which hinders the model’s ability to fully learn and extract key features of missing seedlings during the training process. This may result in the insufficient generalization ability of the model when detecting missing seedlings, thereby reducing the accuracy and robustness of detection. Secondly, the discrepancy between missing and non-missing seedling areas is minimal, thereby impeding the model’s capacity to discern distinctive features during the learning process and consequently affecting the accuracy of detection. Thirdly, human factors, subjective judgment biases, and limitations in the data quality during the manual data annotation process can result in issues such as missing or mislabeled data. These annotation errors can lead to the model learning inaccurate features during training, resulting in detection errors in specific cases. To address this issue, the authors plan to further consider increasing the size of the sample dataset to expand the number of missing seedling samples, adopting more advanced feature extraction methods to enhance the model’s ability to perceive subtle features, and introducing a multi-round manual review mechanism where multiple annotators cross-check the same data to improve the accuracy and consistency of annotations.
(2) The PCERT-DETR model contains 21.4 M parameters and 66.6 G FLOPs, which may result in an increased model inference time. The RT-DETR model utilizes a Transformer architecture, which inherently involves a greater number of parameters and computational costs. Conversely, the results of the ablation experiments suggest that the designed CAFMFusion module adds additional parameters and computational loads. To effectively reduce the number of parameters and computational costs of the RT-DETR model and improve its applicability in resource-constrained environments, future work will consider adopting lighter backbone networks, such as MobileNet, EfficientNet [29], or ShuffleNet [30], to reduce the computational overhead while maintaining strong feature extraction capabilities. Furthermore, the exploration of model compression and acceleration techniques, such as pruning [31], quantization [32], and knowledge distillation, will be undertaken to further optimize the model structure, reduce the computational complexity, and lessen the dependence on high-performance hardware. Additionally, methods like sparse computation, operator fusion, and model architecture optimization will be combined to further reduce the computational cost during model inference and improve the computational efficiency.
(3) The proposed methodology has thus far been evaluated exclusively on a dataset from the early growth stage of rice. Following the training phase of the model, its efficacy was assessed on a dataset comprising 1070 test images. The results indicated that the mAP attained 81.2%, thereby substantiating the proposed methodology’s efficacy for this particular task. Nevertheless, the applicability of this methodology for target detection tasks in the early growth stage of rice remains to be further investigated. In future iterations, the dataset may be expanded to include images from the middle and late growth stages of rice, with the aim of enhancing the model’s capacity to recognize missing seedling areas and improve its adaptability across different growth stages.

## 6. Conclusions

Based on the improved PCERT-DETR model, this study successfully constructed the detection models for the rice seedling stage and missed seedlings. The experimental results show that the mAP of this model on the self-built dataset reaches 81.2%, which is 6.8 percentage points higher than that of the benchmark model RT-DETR, and the F_1_ value is 8.5 percentage points higher, reaching 80.5%. By introducing the PAC-APN module, the hybrid fusion module based on CAFM, and the efficient multi-scale attention mechanism EMA, the detection problems of small morphological differences and strong background interference between rice seedlings and the areas with missing seedlings were effectively solved, and the AP of the missing three seedlings was increased by 20%. With parameters of 21.4 M and a computational efficiency of 66.6 GFLOPs, this model achieved a 98% accuracy rate for seedling recognition and an 81% accuracy rate for detecting the absence of three plants on the test set, significantly outperforming the mainstream YOLO series models.

## Figures and Tables

**Table 1:** The current research status of deep learning for counting and detecting crops.

Reference	Method	Description	Average Precision
[13]	EfficienDet-D1 EfficientNet	Several deep convolutional neural network (DCNN) models were explored. The EfficientDet-D1 EficientNet model performed best in the detection of defective rice seedlings using aerial images.	0.83
[14]	The improved YOLOv8n	This study proposes a lightweight YOLOv8n-based model with HGNetV2 and attention-enhanced modules for robust pepper detection under occlusion and complex backgrounds.	96.3%
[15]	Strawberry R-CNN	A strawberry R-CNN model is proposed. This model can accurately detect and count strawberries and support automatic harvesting and yield estimation.	99.1%, 73.7%
[16]	Yolov5m + BoTNet model	Three CNN models were proposed for the automatic detection of three types of tomato fruits on vines, namely ripe, unripe, and damaged. The modified-Yolov5m-BoTNet model had a high value in the range of 94–96%.	94%
[17]	The optimized YOLOv5s model	This study improves YOLOv5s with optimized network settings and lightweight backbones for accurate, real-time daylily detection across growth stages in complex field conditions, achieving strong performance and robustness.	78.1%
[18]	An enhanced YOLOv9-C model	An improved YOLOv9-C model is proposed for the rapid and accurate detection of mature tomatoes, achieving a mAP of 98%, and has strong applicability to real-world picking tasks.	98%
[19]	YOLOv5-CS mode	A lightweight YOLOv5-CS model is proposed for the rapid and accurate detection of mature apples, which has strong potential in the real-time application of agricultural robots.	99.10%
[20]	MAE-YOLOv8 model	The MAE-YOLOv8 model was proposed for accurate and real-time detection of green and crisp plums, achieving intelligent robot picking in complex orchard environments.	87.5%

**Table 2:** Test environment.

Configuration	Argument
CPU	Intel(R) Xeon(R) Platinum 8352 V CPU @ 2.10 GHz
GPU	NVIDIA GeForce RTX3080 × 2 (20 GB)
Operating system	Ubuntu 20.04
Accelerating environment	Cuda11.3
Development platform	PyCharm
Other	Numpy1.17.0 Opencv4.1.0

**Table 3:** Ablation test.

Test	Baseline	PACAPN	CAFM Fusion	EMA	P (%)	R (%)	F_1_-Score (%)	mAP (%)	Params (M)	FLOPs (G)
1	✓				67.8	77.1	72.0	74.4	19.9	57.0
2	✓	✓			82.9	71.2	76.6	76.6	19.9	58.2
3	✓	✓	✓		84.5	76.2	80.1	78.4	21.4	66.4
4	✓		✓		80.1	73.7	76.8	75.8	21.4	65.1
5	✓			✓	82.7	75.0	78.7	79.6	19.9	57.2
6	✓		✓	✓	75.6	78.5	77.0	77.7	21.5	65.4
7	✓	✓		✓	78.8	78.3	78.5	75.8	19.9	58.4
8	✓	✓	✓	✓	82.8	78.3	80.5	81.2	21.4	66.6

**Table 4:** Comparison of test results of maize seedlings with different models.

Model	P (%)	R (%)	F_1_-Score (%)	mAP (%)	Params (M)	FLOPs (G)
YOLOv8n	82.8	74.4	78.4	78.7	3.1	8.1
YOLOv8m	82.4	76.7	79.4	78.9	25.9	79.1
YOLOv10n	72.8	74.1	73.4	73.0	2.7	8.4
YOLOv10s	69.5	75.0	72.2	75.1	8.1	24.8
YOLOv11n	84.5	72.8	78.2	75.2	2.6	6.3
PCERT-DETR	82.8	78.3	80.5	81.2	21.4	66.6

## Data Availability

The original contributions presented in this study are included in this article; further inquiries can be directed to the corresponding author.

## Abbreviations

AP	Average Precision
CAFM	Convolution and Attention Fusion Module
EMA	Efficient Multi-scale Attention
mAP	Mean Average Precision
UAV	Unmanned Aerial Vehicle
